# Retraction: Regulated programmed cell death in acute lung injury: from pathogenesis to therapy

**DOI:** 10.3389/fimmu.2026.1814313

**Published:** 2026-02-27

**Authors:** Frontiers Editorial Office

The journal retracts the 23 July 2025 article cited above.

Following publication, the authors notified the journal of errors affecting the scientific validity of the article. The results of many of the publications referenced in this review article have been incorrectly applied and cited. An investigation was conducted in accordance with Frontiers’ policies. The authors agree with the retraction as the conclusions have been deemed unreliable.

This retraction was approved by the Chief Editors of Frontiers in Immunology and the Chief Executive Editor of Frontiers. The authors agree to correspondence regarding this retraction.

